# Postoperative survival analysis of hepatocellular carcinoma patients with liver cirrhosis based on propensity score matching

**DOI:** 10.1186/s12893-022-01556-5

**Published:** 2022-03-21

**Authors:** Zhaoqin Wu, Haodong Tang, Lishan Wang, Xiaoling Jin, Zhengqing Lei, Pinghua Yang, Jiahua Zhou

**Affiliations:** 1 Department of Hepatopancreatobiliary Surgery, Zhongda Hospital, School of Medicine, Southeast University, Nanjing, 210009 Jiangsu China; 2 Department of Minimally Invasive Surgery, The Eastern Hepatobiliary Surgery Hospital, Second Military Medical University (Navy Medical University), Shanghai, 200438 China

**Keywords:** Hepatocellular carcinoma, Cirrhosis, Propensity score matching, Overall survival, Recurrence-free survival

## Abstract

**Objective:**

Most hepatocellular carcinoma (HCC) patients in China have some degree of liver cirrhosis. The effect of cirrhosis on the long-term prognosis of HCC patients after hepatectomy is still unclear. This study aimed to investigate the effect of liver cirrhosis on the prognosis of HCC patients after hepatectomy.

**Methods:**

Data from patients who underwent hepatectomy and had pathologically confirmed HCC were retrospectively collected. The patients’ clinical pathological data were recorded. Propensity score matching (PSM) was used to eliminate the influence of potential confounding factors. The Kaplan–Meier method was used to calculate the recurrence-free survival (RFS) and overall survival (OS) rates, and Cox regression analysis was used to screen for independent risk factors affecting OS and RFS.

**Results:**

A total of 1381 HCC patients who were initially treated with hepatectomy were included, including 797 patients with liver cirrhosis. The RFS and OS rates in the group with cirrhosis were significantly lower than those in the group without cirrhosis (after PSM, RFS: P < 0.001; OS: P = 0.001). Subgroup analysis showed that among patients with Barcelona Clinic Liver Cancer (BCLC) stage 0-B disease, RFS and OS were significantly lower in those with cirrhosis than in those without cirrhosis (both P < 0.05); while in patients with stage C disease, there was no significant difference between those with and without cirrhosis. In the group with cirrhosis, alpha-fetoprotein (AFP) > 400, intraoperative blood loss, tumor diameter > 5 cm, satellite lesions, and large vessel invasion were independent risk factors for RFS, while albumin-bilirubin (ALBI) grade, neutrophil-to-lymphocyte ratio (NLR), tumor diameter > 5 cm, satellite lesions, microvascular invasion, and macrovascular invasion were independent risk factors for OS.

**Conclusion:**

HCC with liver cirrhosis has specific characteristics. Compared with patients without cirrhosis, patients with cirrhosis have worse long-term survival after surgery. In addition, the independent risk factors for RFS and OS are different between patients with cirrhosis and without cirrhosis; liver cirrhosis is an independent risk factor for the long-term prognosis of HCC patients, especially patients with BCLC stage 0-B disease after hepatectomy.

## Introduction

Primary liver cancer (PLC) is one of the most common malignant tumors worldwide. In 2020, liver cancer was the sixth most common cancer in the world and the third leading cause of cancer deaths worldwide [1]. Among PLCs, hepatocellular carcinoma (HCC) is the most common type, accounting for 85–90% of PLC cases [2]. According to one report (Globocan), of the 90,600 new cases of HCC reported in the world in 2020, China accounts for more than 50% [3]. Treatments for liver cancer is diverse and require the involvement of multiple disciplines. Among the available treatments, hepatectomy is currently the most important method, and it can enable patients with liver cancer to achieve long-term survival. Liver cancer is a disease with particularly strong clinical and pathological heterogeneity. The long-term survival rates of liver cancer patients with different clinical and pathological characteristics often differ, and the 5-year survival rate ranges from 30 to 70% [4–6] Therefore, early identification of patients at high risk of death after hepatectomy is significant for guiding clinicians to select specific surgical procedures, perioperative nursing strategies, and postoperative follow-up and reexamination approaches for patients with liver cancer.

There are approximately 7 million patients with liver cirrhosis in China [7], of whom 86% have hepatitis B virus-related liver cirrhosis [8]. Most liver cancers develop gradually from chronic hepatitis, cirrhosis, and atypical hyperplastic nodules into liver cancer. At-risk populations should be screened regularly for early detection, early diagnosis, and early treatment [9]. In addition, liver cirrhosis is considered an independent risk factor that affects the prognosis of liver cancer. For HCC patients with liver cirrhosis, hepatic portal occlusion during hepatectomy and retransfusion after massive hemorrhage can easily cause hepatic ischemia–reperfusion injury, thereby affecting their long-term prognosis [10]. In addition, the degree of atypical hepatocyte dysplasia is more severe in HCC patients with cirrhosis and can also cause a poor prognosis after hepatectomy. A retrospective study of 2046 patients enrolled in 10 medical centers in Western and Eastern countries [11] also demonstrated that liver cirrhosis was an independent risk factor that affected long-term survival after HCC surgery [hazard ratio (HR) = 1.41, 95% confidence interval (CI): 1.01–1.95), P = 0.040]. Li et al. [12] found that liver cirrhosis was also an independent risk factor for recurrence after hepatectomy.

The survival-related risk factors for liver cancer patients after hepatectomy mainly comprise three factors: patient factors, tumor factors, and surgical factors. Patient factors include age, sex, history of diabetes, hepatitis, liver cirrhosis, and systemic inflammation indicators; tumor factors include tumor size, number, vascular invasion, capsule integrity, and degree of tumor differentiation; and surgical factors include hepatectomy methods (laparoscopic hepatectomy and open hepatectomy, anatomical hepatectomy and nonanatomical hepatectomy, and distance to the resection margin), intraoperative blood loss, perioperative blood transfusion, etc.

Liver cirrhosis can increase the risk of surgery. The Laennec classification standard is commonly used in clinical practice to divide cirrhosis patients into mild (4A), moderate (4B), and severe (4C) grades to guide treatment and predict their prognosis [13]. The risk of major postoperative complications, including ascites, pulmonary infection, pleural effusion, hepatic encephalopathy, renal failure, portal vein thrombosis, and upper gastrointestinal hemorrhage, is greater in cirrhosis patients than in patients without cirrhosis. This results in a significant increase in perioperative mortality in cirrhotic patients undergoing hepatectomy [14].

Therefore, the survival risk factors for liver cancer patients with and without cirrhosis are different after hepatectomy. This study retrospectively collected the clinical, pathological and follow-up data of HCC patients who underwent hepatectomy to explore the effect of liver cirrhosis on the prognosis of patients with liver cancer after hepatectomy.

## Materials and methods

### Clinical data

Patients who underwent hepatectomy and had postoperative pathological confirmation of HCC at the Zhongda Hospital of Southeast University and the Eastern Hepatobiliary Surgery Hospital, Shanghai, China, between March 2007 and November 2013 were enrolled. The variables collected in this study are related to factors that past studies have suggested may affect the prognosis of HCC after hepatectomy. The variables that were examined in this study included the patient’s region, sex, age, smoking history, drinking history, history of diabetes, antiviral treatment, hepatitis B surface antigen (HBsAg), alpha-fetoprotein (AFP), albumin-bilirubin (ALBI) score, neutrophil-to-lymphocyte ratio (NLR), liver cirrhosis, hepatectomy method, hepatic port occlusion time, surgical blood loss, intraoperative blood transfusion, tumor diameter, number of tumors, satellite lesions, vascular invasion, lymph node invasion, and pathological grading. This study was approved by the ethics committee of the hospitals, and the patients and their families signed informed consent forms.

### Inclusion and exclusion criteria

The inclusion criteria for this study were as follows: 1. Underwent hepatectomy; 2. Postoperative pathological confirmation of HCC; 3. Child–Pugh liver function classification grade A or B; Eastern Cooperative Oncology Group (ECOG) performance status score of 0–2 points; 4. Met the R0 resection criteria. Exclusion criteria: 1. Patients who underwent preoperative transcatheter arterial chemoembolization (TACE), radiofrequency ablation (RFA), microwave ablation (MWA), percutaneous ethanol injection (PEI), or other antitumor therapies; 2. Patients with severe organ dysfunction; 3. Patients with a previous history of other malignant tumors; 4. Patients with perioperative death; 5. Patients with missing clinical pathological data; 6. Patients who were completely lost to follow-up after discharge.

### Follow-up

After discharge, regular outpatient follow-ups or telephone follow-ups were performed. In the first 6 months after surgery, follow-up was performed once every 2 months, then once every 3 months, and once every 6 months after 2 years. The follow-up data included tumor recurrence, metastasis, and survival, which were verified through follow-up by the Jiangsu Provincial Centers for Disease Prevention and Control (CDC). Routine follow-up examinations at outpatient clinics included AFP, liver function, and abdominal ultrasound. If there was a significant increase in AFP during the follow-up period or if ultrasound examination indicated a suspected recurrent nodule, enhanced computed tomography (CT) or magnetic resonance imaging (MRI) of the abdomen was performed. HCC recurrence was considered when imaging revealed typical signs of HCC. Patients with recurrent HCC could be treated with resection alone or with resection combined with TACE, RFA, MWA, PEI, oral sorafenib, or conservative therapy according to the tumor recurrence pattern, the residual liver function reserve capacity, and the patient’s general condition.

The primary endpoint of the study was recurrence-free survival (RFS), which is from the date of hepatectomy for HCC patients to the date of first recurrence, metastasis of intrahepatic or extrahepatic tumors, death, or the date of last follow-up.

The secondary endpoint of the study was overall survival (OS), which is from the date of hepatectomy for HCC patients to the date of death due to tumors or the date of the last follow-up.

### Statistical analysis

Categorical variables were expressed as numbers (n) and proportions (%) and were compared using the Pearson χ^2^ test with Yates continuity correction or Fisher's exact test. Continuous variables were expressed as the mean ± Standard deviation or median (interquartile range, IQR), and the t-test or Mann–Whitney nonparametric U test was used for comparison. The Kaplan–Meier method was used to calculate RFS and OS, Log-rank test was used for comparison between groups. Variables with P values less than 0.05 in the univariate analysis were included in the multivariate Cox proportional hazard regression model to screen independent risk factors. The hazard ratio (HR) and 95% confidence interval (95% CI) were used for statistical description. The difference was considered statistically significant when the P value < 0.05.

## Results

### Comparison of clinicopathological data of HCC patients with and without liver cirrhosis

A total of 1,381 cases were collected according to the inclusion and exclusion criteria of the study. The follow-up date was until April 30, 2019, and the median follow-up time was 84.7 months. Among them, 797 cases were combined with liver cirrhosis. The comparison of clinicopathological data between the patients with liver cirrhosis and those without liver cirrhosis is shown in Table 1. The positive rate of HBsAg was higher in patients with combined cirrhosis (90.1% vs. 79.3%, P < 0.001), and the ALBI score was higher (ALBI ≥ − 2.6, 32.2% vs. 22.1%, P < 0.001). There were also baseline differences between the two groups in age (51.75 years vs. 53.04 years, P = 0.022), history of drinking (21.6% vs. 26.7%, P = 0.032), history of diabetes (21.6% vs. 26.7%, P = 0.032), NLR (NLR ≤ 1.5, 27% vs. 18.7%, P < 0.001), hepatectomy method (anatomical hepatectomy, 51.1% vs. 40.8%, P < 0.001), and tumor diameter (≥ 5 cm, 40.9% vs. 59.8%), and BCLC staging.

**Table 1:** Baseline characteristics before and after PSM in HCC patients with and without cirrhosis

Variables	Before PSM			After PSM		
	Cirrhosis	No cirrhosis	P	Cirrhosis	No cirrhosis	P
	N = 797 (%)	N = 584 (%)		N = 474 (%)	N = 474 (%)	
Gender (%)						
Male	653 (81.9)	489 (83.7)	0.423	386 (81.4)	390 (82.3)	0.8
Female	144 (18.1)	95 (16.3)		88 (18.6)	84 (17.7)	
Age [mean (SD)]	51.75 (10.13)	53.04 (10.63)	0.022*	51.95 (10.04)	52.85 (10.13)	0.168
Smoking (%)	290 (36.4)	233 (39.9)	0.203	169 (35.7)	181 (38.2)	0.459
Drinking (%)	172 (21.6)	156 (26.7)	0.032*	109 (23.0)	108 (22.8)	1
Diabetes (%)	44 (5.5)	49 (8.4)	0.046*	33 (7.0)	31 (6.5)	0.897
HBsAg +, n (%)	718 (90.1)	463 (79.3)	< 0.001**	409 (86.3)	413 (87.1)	0.774
AFP (%)						
≤ 400 μg/L	495 (62.1)	378 (64.7)	0.347	287 (60.5)	310 (65.4)	0.139
> 400 μg/L	302 (37.9)	206 (35.3)		187 (39.5)	164 (34.6)	
MVI (%)						
No	584 (73.3)	428 (73.3)	1	346 (73.0)	347 (73.2)	1
Yes	213 (26.7)	156 (26.7)		128 (27.0)	127 (26.8)	
Prophylactic TACE (%)	319 (40.0)	238 (40.8)	0.828	195 (41.1)	190 (40.1)	0.791
ALBI (%)						
< − 2.6	540 (67.8)	455 (77.9)	< 0.001**	327 (69.0)	371 (78.3)	0.002**
≥ − 2.6	257 (32.2)	129 (22.1)		147 (31.0)	103 (21.7)	
NLR (%)						
≤ 1.5	215 (27.0)	109 (18.7)	< 0.001**	102 (21.5)	97 (20.5)	0.75
> 1.5	582 (73.0)	475 (81.3)		372 (78.5)	377 (79.5)	
Hepatectomy (%)						
Nonanatomic	390 (48.9)	346 (59.2)	< 0.001**	270 (57.0)	268 (56.5)	0.948
Anatomic	407 (51.1)	238 (40.8)		204 (43.0)	206 (43.5)	
Portal triad clamping (%)						
≤ 20 min	610 (76.5)	435 (74.5)	0.416	355 (74.9)	356 (75.1)	1
> 20 min	187 (23.5)	149 (25.5)		119 (25.1)	118 (24.9)	
Intraoperative blood loss (%)						
≤ 400 ml	650 (81.6)	479 (82.0)	0.88	378 (79.7)	393 (82.9)	0.243
> 400 ml	147 (18.4)	105 (18.0)		96 (20.3)	81 (17.1)	
Intraoperative blood transfusion (%)	92 (11.5)	75 (12.8)	0.517	52 (11.0)	61 (12.9)	0.423
Tumor diameter (%)						
< 5 cm	471 (59.1)	235 (40.2)	< 0.001**	228 (48.1)	221 (46.6)	0.696
≥ 5 cm	326 (40.9)	349 (59.8)		246 (51.9)	253 (53.4)	
Number of tumor (%)						
Single	508 (63.7)	367 (62.8)	0.776	292 (61.6)	294 (62.0)	0.947
Multiple	289 (36.3)	217 (37.2)		182 (38.4)	180 (38.0)	
Pathological grading (%)						
I/II	174 (21.8)	115 (19.7)	0.369	89 (18.8)	92 (19.4)	0.869
III/IV	623 (78.2)	469 (80.3)		385 (81.2)	382 (80.6)	
Satellite lesions (%)						
No	635 (79.7)	489 (83.7)	0.065	377 (79.5)	399 (84.2)	0.077
Yes	162 (20.3)	95 (16.3)		97 (20.5)	75 (15.8)	
Macrovascular invasion (%)						
No	690 (86.6)	532 (91.1)	0.012*	431 (90.9)	428 (90.3)	0.824
Yes	107 (13.4)	52 (8.9)		43 (9.1)	46 (9.7)	
Lymphatic metastasis (%)						
No	782 (98.1)	564 (96.6)	0.083	464 (97.9)	456 (96.2)	0.178
Yes	15 (1.9)	20 (3.4)		10 (2.1)	18 (3.8)	
BCLC stage (%)						
0/A	433 (54.3)	233 (39.9)	< 0.001**	222 (46.8)	216 (45.6)	0.745
B/C	364 (45.7)	351 (60.1)		252 (53.2)	258 (54.4)	

*MVI* microvascular invasion, *Prophylactic TACE* prophylactic transcatheter arterial chemoembolization, *ALBI* albumin-bilirubin score, *NLR* neutrophil to lymphocyte ratio

*P < 0.05 **P < 0.01

### Comparison of RFS and OS between HCC patients with and without liver cirrhosis

**Fig. 1:**
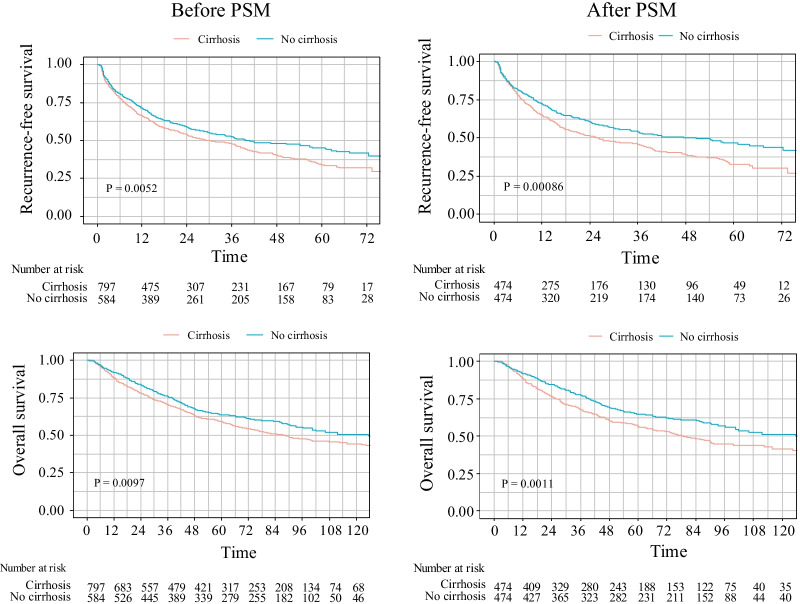
PSM analysis of RFS and OS in HCC patients with and without cirrhosis

The RFS and OS of the group with cirrhosis were significantly lower than those of the group without cirrhosis (Fig. 1). The median RFS in the group with cirrhosis was 30.1 months, and the median RFS in the group without cirrhosis was 39.9 months. The 1-year, 3-year, and 5-year RFS in the group with cirrhosis were significantly lower than those in the group without cirrhosis (66.6% vs. 71.5%, 48.0% vs. 52.2%, 33.9% vs. 45.2%, P = 0.0052). The median OS in the group with cirrhosis was 88.3 months, and the median OS in the group without cirrhosis was 125.7 months. The 1-year, 3-year, 5-year, 10-year OS in the group with cirrhosis were significantly lower than those in the group without cirrhosis (88.6% vs. 91.9%, 70.6% vs. 76.1%, 59.2% vs. 63.6%, 44.3% vs.50.7%, P = 0.0097).

To exclude HBsAg positivity, ALBI, NLR, age, history of drinking, history of diabetes, hepatectomy method, tumor diameter, and large blood vessel invasion that may be confounding factors affecting OS and RFS in the cirrhosis group and the noncirrhosis group, propensity score matching (PSM) analysis was performed to balance the effects of confounding factors that may have an impact on the prognosis of the two groups. PSM analysis was performed by the 1:1-based minimum adjacency method. Survival analysis was performed after balancing the baseline characteristics of the two groups. The baseline characteristics of patients after PSM are shown in Table 1.

We found that after PSM, the RFS and OS of the group with cirrhosis were still significantly lower than those of the group without cirrhosis (Fig. 1). The median follow-up time of the cohort after PSM was 85.4 months, and the median RFS was 26.2 months in the cirrhosis group and 50.6 months in the noncirrhosis group. The 1-year, 3-year, and 5-year RFS in the cirrhosis group were significantly lower than those in the noncirrhosis group (65.1% vs. 72.4%, 46.3% vs. 54.1%, 32.9% vs. 47.0%, P = 0.00086). The median OS in the cirrhosis group was 79.0 months, and the median OS in the noncirrhosis group was 135.0 months. The 1-year, 3-year, 5-year, 10-year OS in the cirrhosis group were lower than that in the noncirrhotic group (88.7% vs. 91.9%, 68.0% vs. 77.7%, 57.1% vs. 64.6%, 41.7% vs. 51.3%. P = 0.0011).

### Subgroup analysis

**Fig. 2:**
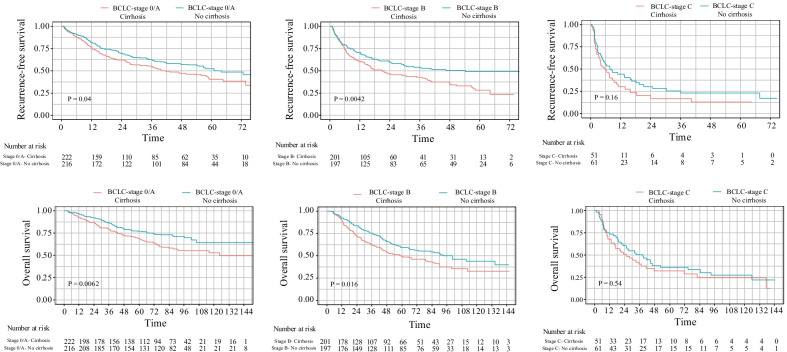
RFS and OS of HCC patients with different BCLC stages of cirrhosis

In this study, all HCC patients were stratified according to BCLC staging. The median RFS was 51.97 months for the stage 0/A, 29.87 months for the stage B, and 5.21 months for the stage C. The 1-year, 3-year, and 5-year RFS rates were 79.7%, 59.4%, and 44.7%; 65.7%, 47.2%, 38.7%, 35.8%, 20.6%, and 18.1%. The median OS could not be estimated for stage 0/A (all survival rates > 50%), 80.5 months in stage B and 25.0 months in stage C. The 1-year, 3-year, 5-year, and 10-year OS rates were 95.3%, 84.1%, 74.2%, 58.7%; 90.8%, 69.5%, 54.8%, 40.0%; 68.3%, 42.2%, and 30.5%, 24.6%, respectively. After PSM, the RFS and OS of BCLC stage A and B patients in the cirrhosis group were significantly lower than those in the noncirrhosis group, and there was no significant difference between the two groups in stage C (Fig. 2).

### Risk factors affecting the long-term prognosis of HCC patients with liver cirrhosis after hepatectomy

The results of the univariate Cox proportional hazard regression model for RFS and OS are shown in Table 2. Univariate analysis showed that AFP > 400 µg/L, intraoperative blood loss > 400 ml, intraoperative blood transfusion, tumor diameter > 5 cm, pathological grade III/IV, satellite lesions, microvascular invasion, and macrovascular invasion were risk factors for RFS and OS in HCC patients with liver cirrhosis after hepatectomy, while female, age, smoking history, and drinking history were risk factors for RFS, NLR > 1.5 and hepatic port occlusion time > 20 min were risk factors affecting postoperative OS.

**Table 2:** Univariate analysis of RFS and OS in HCC patients with and without liver cirrhosis after hepatectomy

Variables	RFS				OS			
	Cirrhosis		No cirrhosis		Cirrhosis		No cirrhosis	
	HR (95% CI)	P	HR (95% CI)	P	HR (95% CI)	P	HR (95% CI)	P
Gender, Female	0.6849 (0.525–0.8935)	0.00526**	0.9562 (0.6888–1.327)	0.789	0.9337 (0.7123–1.224)	0.62	1.364 (0.9753–1.906)	0.0697
Age	0.9907 (0.9816–0.9999)	0.0469*	0.9869 (0.9761–0.9978)	0.0187*	0.9924 (0.9822–1.003)	0.151	0.998 (0.9859–1.01)	0.747
Smoking	1.288 (1.061–1.562)	0.0105*	1.148 (0.9065–1.453)	0.253	1.204 (0.9723–1.49)	0.0888	1.004 (0.771–1.307)	0.978
Drinking	1.431 (1.15–1.782)	0.00135**	1.057 (0.8143–1.372)	0.676	1.145 (0.8962–1.463)	0.279	0.8986 (0.6682–1.208)	0.479
Diabetes	1.017 (0.6841–1.513)	0.932	0.9621 (0.6225–1.487)	0.862	1.477 (0.9974–2.187)	0.0516	0.716 (0.4169–1.23)	0.226
HbsAg( +)	1.425 (0.9989–2.033)	0.0507	1.278 (0.9439–1.731)	0.113	1.034 (0.7357–1.455)	0.845	1.235 (0.8809–1.731)	0.221
AFP, > 400 μg/L	1.649 (1.36–1.998)	< 0.001**	1.378 (1.083–1.754)	0.00918**	1.655 (1.343–2.04)	< 0.001**	1.533 (1.179–1.994)	0.00142**
ALBI, ≥ − 2.6	1.185 (0.9697–1.448)	0.097	1.434 (1.097–1.875)	0.00841**	1.291 (1.041–1.602)	0.0202	1.853 (1.398–2.457)	< 0.001**
NLR, > 1.5	1.194 (0.9601–1.486)	0.111	1.155 (0.8508–1.568)	0.355	1.652 (1.281–2.129)	< 0.001**	1.318 (0.9193–1.889)	0.133
Nonanatomic hepatectomy	1.152 (0.9522–1.393)	0.146	1.159 (0.9156–1.468)	0.22	1.141 (0.9253–1.407)	0.217	0.9993 (0.764–1.307)	0.996
Portal triad clamping time > 20 min	1.137 (0.9116–1.419)	0.254	1.258 (0.9709–1.63)	0.0825	1.32 (1.043–1.67)	0.0208*	1.308 (0.9811–1.743)	0.0673
Intraoperative blood loss > 400 ml	1.6 (1.271–2.015)	< 0.001**	1.27 (0.9418–1.713)	0.117	1.892 (1.489–2.404)	< 0.001**	1.824 (1.353–2.458)	< 0.001**
Intraoperative blood transfusion	1.533 (1.154–2.036)	0.00318**	1.636 (1.185–2.258)	0.00277**	1.807 (1.355–2.41)	< 0.001**	2.097 (1.508–2.916)	< 0.001**
Tumor diameter, > 5 cm	1.986 (1.64–2.405)	< 0.001**	1.489 (1.168–1.898)	0.00129**	2.253 (1.827–2.777)	< 0.001**	2.175 (1.629–2.904)	< 0.001**
Multiple tumors	1.168 (0.961–1.419)	0.119	1.436 (1.134–1.818)	0.00264**	1.173 (0.948–1.451)	0.142	1.468 (1.128–1.91)	0.00424**
Pathological grading III/IV	1.677 (1.308–2.149)	< 0.001**	1.386 (1.015–1.89)	0.0397*	1.681 (1.266–2.232)	< 0.001**	1.965 (1.338–2.885)	< 0.001**
Satellite lesions, Yes	1.729 (1.384–2.161)	< 0.001**	2.022 (1.527–2.678)	< 0.001**	2.027 (1.603–2.563)	< 0.001**	1.913 (1.409–2.596)	< 0.001**
MVI	1.86 (1.52–2.275)	< 0.001**	2.108 (1.653–2.687)	< 0.001**	1.933 (1.553–2.406)	< 0.001**	1.937 (1.474–2.546)	< 0.001**
Macrovascular invasion	3.387 (2.645–4.338)	< 0.001**	3.018 (2.13–4.274)	< 0.001**	2.998 (2.323–3.868)	< 0.001**	2.731 (1.886–3.954)	< 0.001**
Lymphatic metastasis	1.783 (0.9207–3.453)	0.0863	2.159 (1.282–3.636)	< 0.001**	1.74 (0.8973–3.375)	0.101	3.313 (1.958–5.603)	< 0.001**
Prophylactic TACE	1.139 (0.9399–1.381)	0.184	1.036 (0.8171–1.314)	0.769	1.217 (0.9869–1.501)	0.0662	1.097 (0.8434–1.427)	0.49

*P < 0.05 **P < 0.01 *RFS*: recurrence-free survival, *OS* overall Survival, *ALBI* albumin-bilirubin score, *NLR* neutrophil to lymphocyte ratio, *MVI* microvascular invasion, *Prophylactic TACE* prophylactic transcatheter arterial chemoembolization

The above risk factors in the univariate analysis with a P value of < 0.05 were included in the multivariate Cox proportional hazard regression model to explore the independent risk factors for RFS and OS in HCC patients with cirrhosis after hepatectomy. The results are shown in Table 3. In males, AFP > 400, intraoperative blood loss, tumor diameter > 5 cm, satellite lesions, and macrovascular invasion were independent risk factors for RFS. While ALBI, NLR, tumor diameter > 5 cm, satellite lesions, microvascular invasion, and macrovascular invasion were independent risk factors for OS.

**Table 3:** Multivariate analysis of RFS in HCC patients with and without liver cirrhosis after hepatectomy

Variables	**RFS**				**OS**			
	Cirrhosis		No cirrhosis		Cirrhosis		No cirrhosis	
	HR (95% CI)	P	HR (95% CI)	P	HR (95% CI)	P	HR (95% CI)	P
Gender, Female	0.9962 (0.9867–1.0059)	0.4422	–	–	–	–	–	–
Age	0.7328 (0.5493–0.9778)	0.0346*	0.9889 (0.9775–1.00)	0.059165	–	–	–	–
Smoking	1.0829 (0.8525–1.3754)	0.5141	–	–	–	–	–	–
Drinking	1.2703 (0.978–1.65)	0.073	–	–	–	–	–	–
AFP, > 400 μg/L	1.3121 (1.0661–1.6148)	0.0103*	1.097 (0.8434–1.427)	0.489999	1.147 (0.9112–1.444)	0.24273	1.1334 (0.855–1.502)	0.38402
ALBI, ≥ − 2.6	–	–	1.4341 (1.083–1.899)	0.011865*	1.312 (1.0511–1.638)	0.01638*	1.6894 (1.2586–2.268)	< 0.001**
NLR, > 1.5	–	–	–	–	1.372 (1.0532–1.787)	0.01908*	–	–
Portal triad clamping time > 20 min	–	–	–	–	1.023 (0.7954–1.315)	0.86113	–	–
Intraoperative blood loss > 400 ml	1.1844 (0.8741–1.6048)	0.2749	–	–	1.284 (0.9336–1.767)	0.12405	0.8711 (0.5534–1.371)	0.55099
Intraoperative blood transfusion	0.7725 (0.5322–1.1214)	0.1747	1.3469 (0.9524–1.905)	0.092117	0.888 (0.6067–1.3)	0.54136	1.5102 (0.9274–2.459)	0.09752
Tumor diameter, > 5 cm	1.5473 (1.2527–1.9112)	< 0.001**	1.1343 (0.8637–1.49)	0.36473	1.631 (1.2927–2.059)	< 0.001**	1.7445 (1.2685–2.399)	< 0.001**
Multiple tumors	–	–	–	–	–	–	1.009 (0.7229–1.408)	0.9579
Pathological grading III/IV	1.194 (0.9156–1.5571)	0.1905	0.9612 (0.686–1.347)	0.818020	1.175 (0.8665–1.592)	0.29973	1.2592 (0.8359–1.897)	0.27025
Satellite lesions, Yes	1.3558 (1.0718–1.715)	0.0111*	1.5619 (1.158–2.107)	0.003493**	1.742 (1.3648–2.223)	< 0.001**	1.4556 (1.0462–2.025)	0.02588*
MVI	1.2347 (0.9805–1.5549)	0.073	1.8153 (1.3584–2.426)	< 0.001**	1.34 (1.045–1.717)	0.02105*	1.5081 (1.0753–2.115)	0.01729*
Macrovascular invasion	2.2488 (1.6697–3.0289)	< 0.001**	2.0051 (1.3567–2.963)	< 0.001**	1.65 (1.2055–2.26)	0.00177**	1.5668 (1.0402–2.36)	0.03167*
Lymphatic metastasis			2.0317 (1.1955–3.453)	0.008791**	–		3.202 (1.8585–5.516)	< 0.001**

*P < 0.05 **P < 0.01. *RFS* recurrence-free survival, *OS* overall survival, *ALBI* albumin-bilirubin score, *NLR* neutrophil to lymphocyte ratio, *MVI* microvascular invasion, *Prophylactic TACE* prophylactic transcatheter arterial chemoembolization

In HCC patients without cirrhosis after hepatectomy, the univariate Cox proportional hazard regression model showed that age, AFP > 400, ALBI ≥ − 2.6, intraoperative blood transfusion, tumor diameter > 5 cm, multiple tumors, pathological grade III/IV, satellite lesions, microvascular invasion, macrovascular invasion, and lymphatic metastasis are risk factors affecting postoperative RFS, while ALBI ≥ − 2.6, NLR > 1.5, intraoperative blood loss > 400 ml, tumor diameter > 5 cm, multiple tumors, pathological grade III/IV, satellite lesions, microvascular invasion, macrovascular invasion, and lymphatic metastasis were risk factors for postoperative OS. Multivariate analysis suggested that ALBI ≥ − 2.6, satellite lesions, microvascular invasion, macrovascular invasion, and lymphatic metastasis were independent risk factors for postoperative RFS and OS of HCC. In addition, tumor diameter > 5 cm was also an independent risk factor for OS.

## Discussion

Liver cirrhosis is an independent risk factor for RFS and OS of liver cancer, but surgery can improve the OS of liver cancer patients with liver cirrhosis [15]. Our study showed that the postoperative RFS and OS in the HCC group with liver cirrhosis were significantly lower than those of HCC group without liver cirrhosis. After PSM balanced the baseline of the two groups, the postoperative RFS and OS of the HCC group with liver cirrhosis were more significantly lower than those of the HCC group without liver cirrhosis. These results indicate that liver cirrhosis is a major factor affecting RFS and OS in HCC patients. In this study, the PSM method was used for analysis to minimize the selection bias caused by confounding factors and to ensure the balanced comparability of the baseline data between groups.

In this group of HCC patients, some patients with BCLC stage C underwent surgery according to the Chinese guidelines. The OS rates at 1, 3, 5, and 10 years were 68.3%, 42.2%, 30.5%, and 24.6%, respectively. In the subgroup analysis, in BCLC stages A and B, the RFS and OS of cirrhosis patients were worse than those of noncirrhosis patients, while there was no difference between the two in BCLC stage C, indicating that tumor factors were the main factors affecting recurrence in stage C patients rather than the background of liver cirrhosis.

The ALBI score is an important indicator of liver cirrhosis function. The ALBI score of the HCC group with cirrhosis was higher (ALBI ≥ − 2.6, 32.2% vs. 22.1%, P < 0.001), indicating that the liver function reserve of patients with cirrhosis was worse. Previous studies have shown that in patients with solitary HCC with cirrhosis but without macrovascular invasion, tumors larger than 5 cm may significantly affect the prognosis after hepatectomy [16]. Similarly, in this study, tumor diameter > 5 cm was an independent risk factor for RFS and OS in HCC with cirrhosis.

Our study found that female was a protective factor for RFS in the cirrhosis group. As an independent risk factor for RFS, satellite lesions and macrovascular were the same in the two groups. However, AFP > 400, intraoperative blood loss, and tumor diameter > 5 cm were independent risk factors for HCC with cirrhosis only. While ALBI ≥ − 2.6, microvascular invasion, and lymphatic metastasis were the independent risk factors for HCC without cirrhosis only. Multivariate analysis of OS showed that ALBI ≥ − 2.6, tumor diameter > 5 cm, satellite lesions, microvascular invasion, and macrovascular invasion were all independent risk factors for the two groups. NLR was an independent risk factor for the HCC group with cirrhosis only. Lymphatic metastasis was an independent risk factor for HCC without cirrhosis only.

## Conclusion

In summary, liver cancer with liver cirrhosis has its own characteristics and poor liver reserve function. Compared with patients without cirrhosis, patients with cirrhosis have worse long-term survival after surgery. In addition, the independent risk factors for RFS and OS are different between patients with cirrhosis and without cirrhosis; liver cirrhosis is an independent risk factor for the long-term prognosis of HCC patients, especially patients with BCLC stage 0-B disease after hepatectomy. Therefore, it is necessary to carry out comprehensive perioperative nursing intervention and routine nursing intervention for liver cancer patients with liver cirrhosis, treat postoperative stress reaction in liver cancer patients with cirrhosis to shorten the recovery process and strengthen the follow-up after discharge.

## Data Availability

The datasets used and/or analysed during the current study are available from the corresponding author on reasonable request.

## Abbreviations

AFP: Alpha-fetoprotein
ALBI: Albumin-bilirubin score
BCLC: Barcelona Clinic Liver Cancer (BCLC) stages
CI: Confidence interval
HBsAg: Hepatitis B surface antigen
HBV: Hepatitis B virus
HCC: Hepatocellular carcinoma
HR: Hazard ratio
MVI: Microvascular invasion
MWA: Microwave ablation
NLR: Neutrophil to lymphocyte ratio
OS: Overall Survival
PEI: Percutaneous ethanol injection
PSM analysis: Propensity score matching analysis
RFA: Radiofrequency ablation
RFS: Recurrence-free survival
TACE: Transcatheter arterial chemoembolization
